# Outcomes and other characteristics of the nutritional screening and assessment tools validation processes in hospitalized adults: a systematic review

**DOI:** 10.31744/einstein_journal/2026RW1855

**Published:** 2026-04-24

**Authors:** Giovanna Guimarães Lopes Bergamasco, Bruna Rodrigues Lima, Kadine Andrade de Araújo, Nicolly Baião Martins, Silvia Maria Fraga Piovacari, Mayumi Shima, Drielle Schweiger Freitas Bottairi, Samara Cristina Mascarenhas Ferreira, Zelita Melo Fortes de Cerqueira, Adriano José Pereira

**Affiliations:** 1 Hospital Israelita Albert Einstein Clinical Nutritional Department São Paulo SP Brazil Clinical Nutritional Department, Hospital Israelita Albert Einstein, São Paulo, SP, Brazil.; 2 Hospital Israelita Albert Einstein Faculdade Israelita de Ciências da Saúde Albert Einstein São Paulo SP Brazil Postgraduate Students, Faculdade Israelita de Ciências da Saúde Albert Einstein, Hospital Israelita Albert Einstein, São Paulo, SP, Brazil.; 3 Hospital Israelita Albert Einstein Faculdade Israelita de Ciências da Saúde Albert Einstein São Paulo SP Brazil Faculdade Israelita de Ciências da Saúde Albert Einstein, Hospital Israelita Albert Einstein, São Paulo, SP, Brazil.; 4 Hospital Israelita Albert Einstein E-GATE (Einstein-Global Advanced Technologies for Equity) São Paulo SP Brazil E-GATE (Einstein-Global Advanced Technologies for Equity), Hospital Israelita Albert Einstein, São Paulo, SP, Brazil.

**Keywords:** Nutrition assessment, Malnutrition, Risk factors, Screening

## Abstract

**Background:**

Hospital malnutrition has been studied for decades; however, its prevalence remains high, and research in this area is still relevant. Nutritional screening and assessment tools are routinely used in hospital settings.

**Objective:**

We aimed to describe and discuss the general characteristics of studies that used nutritional screening and assessment instruments in hospitalized adult populations, with a focus on clinical outcomes.

**Methods:**

We conducted a systematic review without meta-analysis. Eligible studies were original prospective or retrospective studies published in Portuguese or English, with no inception date, conducted in hospitalized adult populations, and reporting clinical outcomes. Information sources included PubMed, LILACS, Web of Science, Embase, and Scopus. The search covered articles published up to July 2022. Risk of bias was assessed for all included studies using the Quality Assessment of Diagnostic Accuracy Studies-2 (QUADAS-2) tool. Results presented in the tables were transcribed from the main findings of each evaluated study, and no new statistical analyses were performed.

**Results:**

Seventy-seven studies were included, encompassing 20 tools evaluated in hospital settings. The Mini Nutritional Assessment was the most extensively tested in relation to clinical outcomes. Among studies conducted in Brazilian populations, the nutritional assessment tool Subjective Global Assessment and the screening tool Nutritional Risk Screening 2002 were the most frequently studied. Among full-text articles assessed for tool evaluation in hospitals, 77% were excluded because clinical outcomes were not reported.

**Discussion:**

The evidence has some limitations. First, the exclusive focus on hospitalized populations may limit generalizability. Second, validation studies that did not evaluate clinical outcomes were not described in detail. Third, the use of "clinical outcomes" as a search term may have led to an underestimation of validation studies focusing solely on diagnostic or screening performance. Overall, nutritional screening and assessment tools commonly used in daily practice have been validated many years ago, largely based on subjective clinical assessments, with relatively few studies reporting clinical outcomes. In addition, most studies were single-center and conducted predominantly in non–Latin American populations. Future research should prioritize multicenter designs, improve population representativeness, and incorporate clinically relevant outcomes.

**Prospero database registration:**

ID CRD42022347507.

## INTRODUCTION

Hospital malnutrition has been studied for decades; however, its prevalence remains high, highlighting the continued importance of research in this field.^(1,2)^ Hospital malnutrition is associated with poor prognosis, a high risk of complications, functional impairment, increased morbidity and mortality, prolonged hospital stays, and high readmission rates.^(3)^ In addition, malnutrition imposes a substantial economic burden on healthcare systems.^(4,5)^

Nutritional screening is an essential first step in identifying patients at nutritional risk and should be performed at hospital admission by nutritionists or other healthcare professionals.^(6-9)^ Screening should be quick and straightforward and can be completed by any trained healthcare professional within 24–48 hours of admission.^(6-9)^ Patients identified as being at nutritional risk should then undergo a comprehensive nutritional assessment; those not at risk may be rescreened within 7 days. Despite their clinical importance, many nutritional screening tools currently in use were validated approximately two decades ago.^(10-12)^

Nutritional assessment is a systematic and comprehensive process that integrates nutritional and clinical information and provides the basis for planning nutrition interventions.^(13,14)^ In hospital practice, validated tools are commonly used for assessment, although they are often regarded as semi–gold standards.^(13,14)^ Key limitations include the lack of a single standard tool and heterogeneity in assessed parameters, validation methods, and reported outcomes, which may contribute to misclassification of malnutrition.^(14,15)^Notably, some widely used nutritional tools were originally validated in retrospective studies and/or relied primarily on subjective parameters or laboratory tests.^(12,16)^

## OBJECTIVE

We aim to describe and discuss the general characteristics of studies that validated nutritional screening and assessment instruments in hospitalized adult populations, with a focus on clinical outcomes.

## METHODS

This systematic review was conducted without meta-analysis and was reported in accordance with the Preferred Reporting Items for Systematic Reviews and Meta-Analyses (PRISMA) guidelines.^(17)^

### Eligibility criteria

We included original prospective or retrospective studies published in Portuguese or English, with no restriction on inception date, conducted in hospitalized adult human populations, and reporting clinical outcomes. We excluded reviews, editorials, and case reports.

### Information sources

We searched PubMed, LILACS, Web of Science, Embase, and Scopus for articles published up to July 2022.

### Search strategy

The main search strategy used in this review (initiated with a PubMed/MEDLINE search) is described below: (((("malnutrition"[MeSH Terms]) AND ((("mass screening"[MeSH Terms]) OR ("nutrition assessment"[MeSH Terms])))) AND (("physical functional performance"[MeSH Terms]) OR ("treatment outcome"[MeSH Terms]))) OR ((((((((("mass screening"[Title/Abstract]) OR ("nutrition assessment"[Title/Abstract])) OR ("nutritional risk screening"[Title/Abstract])) OR ("mini nutritional assessment short form"[Title/Abstract])) OR ("sga"[Title/Abstract])) OR ("subjective global assessment"[Title/Abstract])) OR ("mini nutritional assessment"[Title/Abstract])) AND ((("malnutrition"[Title/Abstract]) OR ("malnourish"[Title/Abstract])) OR ("malnourished"[Title/Abstract]))) AND (("physical functional performance"[Title/Abstract]) OR ("functionality"[Title/Abstract])))) OR ((((((((("mass screening"[Text Word]) OR ("nutrition assessment"[Text Word])) OR ("nutritional risk screening"[Text Word])) OR ("mini nutritional assessment short form"[Text Word])) OR ("sga"[Text Word])) OR ("subjective global assessment"[Text Word])) OR ("mini nutritional assessment"[Text Word])) AND ((("malnutrition"[Text Word]) OR ("malnourish"[Text Word])) OR ("malnourished"[Text Word]))) AND (("physical functional performance"[Text Word]) OR ("functionality"[Text Word]))).

### Selection process

Three independent researchers systematically identified studies, with support from a librarian from the *Instituto de Ensino e Pesquisa Albert Einstein*. Study selection was managed using Rayyan (Cambridge, MA, USA; 2023).^(18)^ Titles and abstracts were screened against the inclusion criteria. Disagreements were resolved through reassessment and discussion in a formal consensus meeting.

### Data collection process and data items

For studies included in the full-text analysis, we extracted the following information: country, study design, population, place of hospitalization, diagnosis, tool(s) evaluated, comparators, number of centers, inclusion criteria, sample size, outcomes/metrics, and main results. Data were transcribed directly from the included articles to minimize interpretation bias. Study design (prospective vs retrospective) was not always explicitly reported; in such cases, we classified design based on the described methodology for the purposes of tabulation. When variables were not clearly reported, they were recorded as "not specified."

### Study risk bias, reporting bias, and certainty assessment

Risk of bias was assessed for all included studies using the Quality Assessment of Diagnostic Accuracy Studies-2 (QUADAS-2) tool. A calibration meeting was held before assessment to standardize interpretation of each domain. Five authors conducted the assessments independently, and two authors subsequently reviewed all ratings in full, including one reviewer who had not participated in the initial assessments. Discrepancies were resolved by consensus. All authors reviewed the final report after manuscript completion.

### Effect measures

Because the data were not suitable for meta-analysis, only descriptive analyses were performed.

### Synthesis methods

We verified eligibility by confirming study design, publication language, and population characteristics (human adults hospitalized in a hospital setting) based on the Methods sections of the included articles. The results presented in the tables were transcribed from the main findings of each evaluated study, and no new statistical analyses were performed. References were managed using Mendeley (Mendeley Ltd., United Kingdom; version 2.80.1; 2022).

## RESULTS

### Study selection

**Figure 1:**
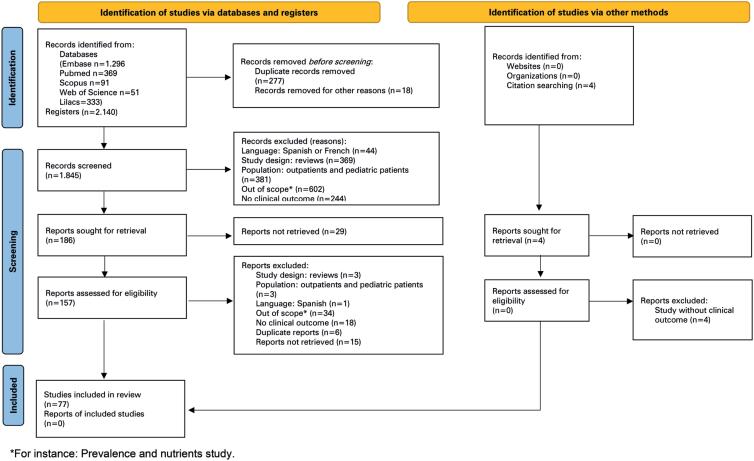
PRISMA flow diagram of study selection

A total of 2,140 articles were found by the search strategy. After peer analysis and evaluation, 77 studies were included in the final analysis. Figure 1 provides the flowchart of article selection and the reasons for exclusions.

### Study characteristics

Among the articles eligible for the evaluation of nutritional tools, 77% were excluded from the analysis because clinical outcomes were not considered in any of the analyses. The results below detail the 77 studies included because they used clinical outcomes in testing nutritional tools.

The included studies involved populations from 31 countries. Twenty-one studies (28%) had a prospective observational design, and 19 (25%) were prospective cohort studies. Overall, 77% were conducted in single-center settings, while 18% were multicenter studies. In 30 studies, only populations aged >60 years were evaluated. Most studies included clinical and surgical patients (90%).

Regarding outcomes, 51 studies (66%) assessed mortality, and 34 assessed length of hospital stay (44%). Functional outcomes, such as frailty and functional capacity, were evaluated in only six studies (8%).

Overall, 20 tools were tested in the hospital setting: Mini Nutritional Assessment (MNA), Mini Nutritional Assessment Short Form (MNA-SF), Nutritional Risk Screening 2002 (NRS 2002), Malnutrition Universal Screening Tool (MUST), Malnutrition Screening Tool (MST), Modified Nutrition Risk in Critically Ill (m-NUTRIC), Nutrition Risk in Critically Ill (NUTRIC), Subjective Global Assessment (SGA), Patient-Generated Subjective Global Assessment (PG-SGA), Geriatric Nutritional Risk Index (GNRI), Malnutrition-related Complications Score (MCRS), Automated Nutrition Score (ANS), Prognostic Nutritional Index (PNI), Controlling Nutritional Status (CONUT), Onodera's Prognostic Nutritional Index (O-PNI), Short Nutritional Assessment Questionnaire (SNAQ), Royal Free Hospital-Nutritional Prioritizing Tool (HFR-TNP), 3-Minute Nutrition Screening (3-MinNS), European Society for Clinical Nutrition and Metabolism Diagnostic Criteria for Malnutrition (ESPEN-DCM), and Academy of Nutrition and Dietetics-European Society for Clinical Nutrition and Metabolism Criteria (AND-ESPEN). Studies evaluating the Global Leadership Initiative on Malnutrition (GLIM) criteria in combination with tools were also included.

### Risk of bias in studies:

**Figure 2:**
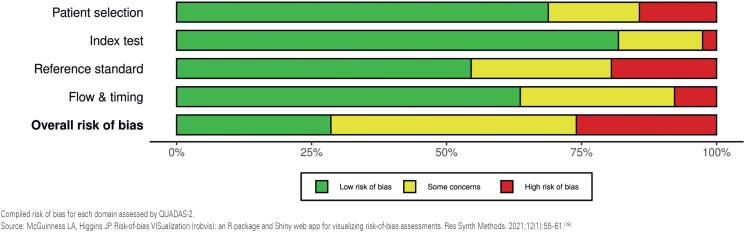
Summary of risk of bias for each domain and overall risk in the included studies assessed using QUADAS-2

**Figure 3:**
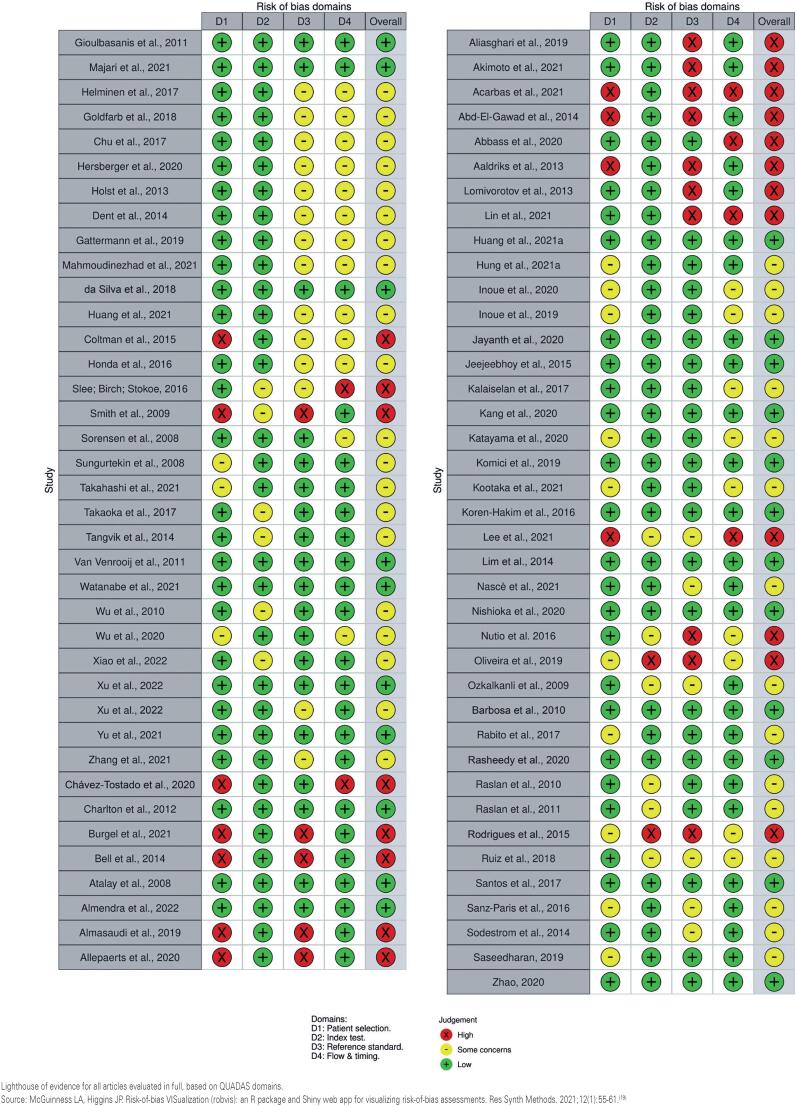
Risk of bias summary for each included study assessed using QUADAS-2

Risk of bias is summarized in figure 2 and presented by article in figure 3. Overall, approximately 30% of studies were rated as low risk of bias, 45% as some concerns, and 25% as high risk.

### Results of individual studies:

The comprehensive analysis of the retrieved articles is presented in table 1. Table 2 provides a summary of the tools discussed, along with their respective outcomes.

**Table 1:** Characteristics and main results of the included studies

Article	Country	Study design	Population	Population: place of hospitalization	Population; Diagnosis	Tool	Comparator	Center	Inclusion criteria	Sample size	Outcome	Main results
Gioulbasanis et al.^(20)^ 2011	Greece	Retrospective, observational study	Metastatic lung cancer prior to commencement of systemic therapy	Clinical	Oncological	MNA	≥5% weight loss (independent variable)	Single-center	>18 years	171	Prognostic prediction: time to disease progression and overall survival	The MNA better predicts all evaluated outcomes. Malnourished individuals identified by MNA exhibit a reduced average survival of 2.07 months
Majari et al.^(21)^ 2021	Iran	Prospective cohort study	Critically ill patients	ICU	Neurological; gastrointestinal/ hepatic; cardiac; malignant diseases	MUST	M-NUTRIC and NRS 2002	Multicenter	>18 years	440	Length of hospital stay; duration of mechanical ventilation; 28-day mortality	No association between M-NUTRIC or NRS scores and evaluated outcomes
Helminen et al.^(22)^ 2017	Finland	Prospective cohort study	Elderly individuals with hip fracture	Surgical	Hip fracture	MNA-SF	MNA complete	Single-center	≥65 years	594	Mobility at 1 and 4 months; quality of life at 1 year; mortality at 1 and 4 months and at 1 year after fracture	Both tools predicted mortality at all timepoints and predicted mobility and quality of life at 4 months
Goldfarb et al.^(23)^ 2018	Canada, USA, and France	Prospective cohort study	Elderly individuals undergoing cardiovascular surgery	Surgical	Cardiovascular disease	MNA-SF	N/A	Multicenter	≥70 years	1,158	1-year mortality; 30-day mortality and morbidity; frailty	Patients classified as malnourished by MNA had a threefold higher risk of 1-year mortality. A moderate association was observed between MNA score and frailty (SPPB)
Chu et al.^(24)^ 2017	China	Prospective observational study	Elderly individuals undergoing elective orthopedic surgery	Surgical	Orthopedic	MNA-SF	N/A	Single-center	≥60 years	312	Mortality at 12 Months Hospital Readmission, Emergency Department Visits, and Functionality	Lower MNA-SF scores associated with increased emergency department visits at 6 months and higher mortality at 12 months. No association was observed with hospital readmission or functional decline. Each 1-point increase in MNA score was associated with a 27% decrease in mortality risk
Hersberger et al.^(25)^ 2020	Switzerland	Retrospective observational study	Medical inpatient units	Clinical	Mixed hospital population	NRS 2002	n/a	Multicenter	Adults with NRS ≥3 points and expected LOS >4 days	2,028	Mortality at 30 to 180 days ICU admission, hospital readmission and complications at 30 to 180 days	NRS is an independent predictor of mortality and incidence of adverse events at 180 days. NRS 2002 is associated with an ↑ average length of hospital stays
Holst et al.^(26)^ 2013	Denmark and Sweden	Prospective cohort study	Hospitalized elderly individuals	Clinical/Surgical	Gastrointestinal and neurological diseases; orthopedic	MNA, MUST, and NRS 2002	Anthropometry and Cognitive Test (SPMSQ)	Multicenter	>65 years	233	12-month mortality; anthropometric assessment	No significant association between the three tools and mortality
Dent et al.^(27)^ 2014	Australia	Cross-sectional study	Hospitalized elderly individuals in the geriatric ward	Clinical	Uninformed	GNRI, MNA e MNA-SF	Incorporation of calf circumference or BMI into the tools	Single-center	Elderly	172	6-month mortality; readmission/transfer to higher level of care	Full MNA and GNRI predicted adverse outcomes at 6 months better than the MNA-SF versions
Gattermann al.^(28)^ 2019	Brazil	Prospective observational study	Critically ill surgical patients	ICU	Gastrointestinal and cardiovascular surgeries	SGA	Clinical assessment	Single-center	≥18 years	76	In-hospital mortality; Hospital LOS; ICU LOS	No significant correlation was observed with mortality. Patients classified as malnourished by SGA had a 2.5-fold higher risk of longer hospitalization
Mahmoudinezhad et al.^(29)^ 2021	Iran	Prospective cross-sectional study	Stroke and LOS >48 h.	Clinical	Neurologic	SGA	N/A	Multicenter	≥65 years	349	Functional capacity	A significant association was observed between ≥3 levels of disability and SGA
da Silva et al.^(30)^ 2018	Brazil	Prospective observational study	Patients admitted to the emergency department	Clinical	Mixed hospital population	ESPEN-DCM	SGA, MUST, NRS 2002, SNAQ, and MST	Single-center	≥18 years	750	LOS; incidence of hospital-acquired infection; in-hospital mortality	NRS 2002 showed a better correlation with mortality. ESPEN-DCM had low diagnostic value but performed better when combined with NRS for prognostic prediction
Huang et al.^(31)^ 2021	China	Prospective cohort study	Cancer patients undergoing radical gastrectomy	Surgical	Oncological	GLIM (screened with NRS 2002)	Isolated criteria vs combination (1 phenotypic + 2 etiological factors)	Single-center	≥65 years	597	Overall survival; disease-free survival	The combined GLIM criteria outperformed isolated factors and was significantly associated with overall and disease-free survival
Coltman et al.^(32)^ 2015	USA	Prospective observational study	Patients admitted to medical, surgical, and neurology intensive care units	ICU	Clinical and surgical diagnoses	Regional Institutional Nutritional Screening Tool	SGA and NUTRIC	Single-center	≥18 years	294	LOS ICU mortality	NUTRIC, alone or in combination, improved the identification of mortality risk. The tools did not predict risk in patients with a long LOS, suggesting limited suitability for this population
Honda et al.^(33)^ 2016	Japan	Prospective cohort study	Patients with acute heart failure	Clinical	Cardiovascular disease	GNRI	N/A	Single-center	≥65 years	490	Mortality (cardiovascular and all-cause)	GNRI was an independent predictor of adverse events in patients with acute heart failure
Slee et al.^(34)^ 2016	UK	Retrospective cohort study	Frail elderly individuals receiving nutrition orally or via a feeding tube.	Clinical	Not specified	MUST	MNA-SF	Single-center	>65 years	78	Mortality; LOS	MNA-SF was a significant predictor of mortality, whereas MUST showed a weak association
Smith et al.^(35)^ 2009	Australia	Prospective cohort study	Surgical inpatients	Surgical	Gastrointestinal diseases	SGA	MRCS and ANS	Single-center	Not specified	148	Risk of postoperative complications	MRCS had a higher predictive value for postoperative complications than SGA
Sorensen et al.^(36)^ 2008	Denmark	Prospective cohort study	Hospitalized adults	Clinical/Surgical	Gastrointestinal diseases, oncological; critical care	NRS 2002	N/A	Multicenter	≥18 years	5,051	Hospital complications; mortality; LOS	NRS 2002 was an independent predictor of all evaluated outcomes
Sungurtekin et al.^(37)^ 2008	Turkey	Prospective observational study	Critically ill adults	ICU	Surgical	SGA	N/A	Single-center	≥18 years	124	ICU mortality; ICU LOS	SGA was able to predict the evaluated outcomes
Takahashi et al.^(38)^ 2021	Japan	Retrospective cohort study	Patients with completely resected non-small cell lung cancer	Surgical	Oncological	GNRI	PNI and CONUT	Single-center	Not specified	475	Overall Survival Postoperative Complication	PNI, CONUT, and GNRI showed high predictive value for the evaluated outcomes
Takaoka et al.^(39)^ 2017	Japan	Retrospective observational study	Patients with crohn's disease admitted to gastroenterology	Clinical	Gastrointestinal Diseases	SGA	NRS 2002, O-PNI, MUST and CONUT	Single-center	Not specified	40	LOS >28 days	All tested tools, except for MUST, were associated with length of stay. SGA was able to predict the studied outcomes
Tangvik et al.^(40)^ 2014	Norway	Prospective observational study	Inpatients	Clinical/Surgical/ICU	Uninformed	NRS 2002	Adapted NRS 2002 (using only the first four questions)	Single-center	≥18 years	3,279	Mortality; morbidity; readmission	The adapted NRS proved to be a strong predictor of the evaluated outcomes
van Venrooij et al.^(41)^ 2011	Netherlands	Cohort study	Patients undergoing coronary artery graft surgery admitted to the cardiothoracic surgical ward	Surgical	Cardiovascular disease	MUST	MUST modified for cardiovascular cirurgical and SNAQ	Single-center	≥18 years	325	Adverse postoperative outcomes	Modified MUST for cardiac surgery was associated with longer ICU and hospital stays
Watanabe et al.^(42)^ 2021	Japan	Retrospective observational study	Elderly people with renal cell cancer	Surgical	Oncological	GNRI	N/A	Single-center	≥65 years	62	Postoperative complications	Patients with postoperative complications had significantly lower GNRI values
Wu et al.^(43)^ 2010	China	Prospective observational study	Patients with newly diagnosed gastrointestinal cancer (without metastasis) undergoing surgery	Surgical	Oncological	SGA	N/A	Single-center	≥18 years	505	LOS complications and hospital cost prediction	SGA classification was significantly associated with LOS and costs but not with complications
Wu et al.^(44)^ 2020	China	Prospective observational study	Patients with cirrhosis without liver cancer or uncontrolled comorbid disease	Clinical	Liver diseases	HFR-TNP	NRS, MUST, and HFR-TNP	Single-center	Adults	155	Mortality	MUST showed lower sensitivity. HFR-TNP predicted outcomes better than NRS 2002
Xiao et al.^(45)^ 2022	China	Retrospective observational study	Patients undergoing radical gastrectomy	Surgical	Oncological	CONUT	N/A	Single-center	≥18 years	106	Postoperative complications; quality of life; overall survival	CONUT was an independent risk factor for postoperative complications and quality of life. Higher CONUT scores were associated with shorter survival
Xu et al.^(46)^ 2022	China	Retrospective cohort study	Patients with primary gastric adenocarcinoma before surgery	Surgical	Oncological	GLIM	Phenotypic criteria	Single-center	Not specified	1,188	Survival time; postoperative complication	Weight loss showed the strongest correlation with survival and complications. Greater weight loss and low muscle mass (tomography) were associated with worse outcomes
Xu et al.^(47)^ 2022	China	Retrospective analytics study	Patients with gastric cancer undergoing radical gastrectomy	Surgical	Oncological	GLIM with MUST screening score ≥1	SGA	Single-center	Not specified	895	Postoperative complications at 1 month; In-hospital mortality; LOS readmission; mortality	GLIM criteria showed a moderate association with SGA. Patients classified as malnourished by GLIM were predictors of survival and complications
Yu et al.^(48)^ 2021	China	Retrospective observational study	Patients with cardiovascular disease(acute coronary syndrome)	Surgical	Cardiovascular disease	NRS-2002	N/A	Multicenter	Not specified	3,185	Mortality	NRS ≥3 points associated with mortality and the incidence of acute kidney disease in patients with ACS
Zhang et al.^(49)^ 2021	China	Prospective observational study	Patients with cardiovascular disease and primary diagnosis of stroke	Clinical	Cardiovascular and neurological diseases	ESPEN-DCM	CONUT GNRI, MUST and NRS-2002	Multicenter	≥18 years	593	Mortality at 3 and 12 months after discharge; functional capacity (mRS)	NRS 2002 had better predictive value for mortality at 3 and 12 months. NRS-2002 and MUST were significantly associated with mRS at 12 months after discharge
Chávez-Tostado et al.^(50)^ 2020	Mexico	Prospective cross-sectional study	Patients hospitalized with gastrointestinal disease(excluding ICU)	Clinical	Gastrointestinal disease	NRS-2002, SGA e CONUT	N/A	Single-center	18-90 years	196	LOS; complications; mortality	CONUT was the only tool to predict complications. None of the tools predicted mortality
Charlton et al.^(51)^ 2012	Australia	Retrospective observational study	hospitalized	Clinical	Uninformed	MNA	N/A	Multicenter	>65 years	2,076	Readmission; LOS; mortality	Nutritional status assessed by MNA at admission was correlated with LOS
Burgel et al.^(52)^ 2021	Brazil	Prospective observational study	hospitalized, expect icu	Clinical/Surgical	Uninformed	AND-ASPEN	SGA	Multicenter	≥18 years	600	Mortality; readmission; LOS	AND-ASPEN predicted an approximately 1.4-fold greater risk of prolonged hospitalization and hospital readmission. In addition to identifying a 5.0 times. higher risk of death in hospital and within 6 months, in an adjusted analysis
Bell et al.^(53)^ 2014	Australia	Accuracy study	hip fracture	Surgical	Hip fracture	MNA-SF	CID-10AM (malnutrition)	Single-center	Elderly	142	Postoperative mobility; mortality; home discharge	MNA-SF predicts probability of home discharge and mortality within 4 months, but not reduction in postoperative mobility
Atalay et al.^(54)^ 2008	Turkey	Retrospective observational study	critically ill elderly hospitalized with enteral and parenteral nutritional therapy	Clinical	Neurological, cardiovascular and oncological diseases	SGA	N/A	Single-center	≥65 years	119	Mortality; LOS	SGA classification did not influence the mortality rate
Almendra et al.^(55)^ 2022	Brazil	Retrospective observational study	elderly	Clinical	Gastrointestinal, renal and oncological diseases	MNA	NRS-2002 and MNA	Single-center	≥65 years	277	LOS	MNA was the tool associated with length of hospital stay
Almasaudi et al.^(56)^ 2019	UK	Retrospective observational study	patients undergoing surgery for colorectal cancer	Surgical	Oncological	MUST	N/A	Single-center	Not defined	363	Postoperative complications; LOS; mortality	MUST demonstrated to be an independent marker for length of hospital stay, but not for complications Medium and high risk by MUST demonstrated higher mortality
Allepaerts et al.^(57)^ 2020	Belgium	Prospective cross-sectional study	Patients admitted to geriatrics	Clinical	Neurological, oncological diseases	GLIM with MNA-SF screening	a phenotypic and an etiological criterion	Single-center	Eldery	79	1-year institutionalization; mortality	GLIM (one phenotypic and one etiological criterion) was associated with 1-year mortality
Alisgahari et al.^(58)^ 2019	Iran	Cross-sectional study	Patients with ischemic stroke	Clinical	Neurological diseases	MNA	N/A	Multicenter	≥65 years	253	Biochemical and anthropometric parameters; LOS; mRS Performance	MNA was significantly associated with poor outcomes in this subgroup
Akimoto et al.^(59)^ 2021	Japan	Retrospective observational study	Patients with ischemic stroke	Clinical	Neurological diseases	CONUT	GNRI	Single-center	≥65 years	218	Anthropometric parameters; LOS; mRS Performance	CONUT was associated with poorer prognosis, higher mRS scores, and more complications
Acarbaş^(60)^ 2021	Turkey	Retrospective observational study	Patients undergoing spinal surgery	Surgical	Spinal surgery	PNI, CONUT and GNRI	N/A	Single-center	≥65 years	454	Prediction of perioperative adverse events.	Higher CONUT scores (pre-surgical) were associated with adverse events. GNRI showed the greatest accuracy for this outcome
Abd-el-gawad et al.^(61)^ 2014	Egypt	Prospective cohort study	Patients admitted to geriatrics	Clinical	Not specified	MNA	GNRI	Single-center	≥60 years	131	LOS; Infection-related complications; mortality	GNRI was an independent predictor of mortality at 3 and 6 months
Abbass et al.^(62)^ 2020	Scotland	Retrospective observational study	Patients with lung cancer prior to radiotherapy	Clinical	Oncologic	MUST	Modified frailty index (mFI)	Single-center	Not specified	643	Overall survival	MUST was independently associated with overall survival and was a prognostic indicator of 12-month survival
Aaldriks et al.^(63)^ 2013	Netherland	Prospective observational study	Patietns with colorectal cancer prior to chemotherapy	Clinical	Oncologic	MNA	Cognitive decline, frailty and mental status questionnaires	Multicenter	≥70 years	143	Mortality	MNA was associated with mortality and predicted poor tolerance of chemotherapy cycles
Lomivorotov et al.^(64)^ 2013	Russia	Prospective observational study	Patients undergoing extracorporeal circulation	Surgical	Cardiovascular disease	SGA, NRS-2002, MUST, MNA-SF and SNAQ	N/A	Single-center	Adults	1,193	Mortality; postoperative complications; LOS; ICU LOS	Only MUST and MNA-SF were independent predictors of postoperative complications. SNAQ, MUST, and MNA predicted prolonged hospitalization
Lin et al.^(65)^ 2021	China	Retrospective observational study	Patients with tuberculosis	Clinical	Tuberculosis	PG-SGA	N/A	Single-center	≥65 years	128	Mortality Liver injury	High PG-SGA scores were independently associated with mortality and liver injury
Huang et al.^(66)^ 2021	China	Prospective observational study	Overweight patients undergoing radical gastrectomy for gastric cancer	Surgical	Oncologic	GLIM	Addition of muscle strength and gait speed variables	Single-center	Not specified	587	Postoperative complications Mortality	Adding muscle quality (tomography), strength (handgrip), and gait speed (6 m) to GLIM improved prediction of outcomes, with gait speed showing the strongest contribution
Hung et al.^(67)^ 2021	Taiwan	Prospective cohort study	Patients with head and neck cancer undergoing concomitant chemoradiotherapy	Clinical	Oncologic	MNA-SF	N/A	Multicenter	≥20 years	461	Overall survival	MNA-SF was correlated with toxicity and adverse events and was associated with a lower hospital readmission rate
Inoue et al.^(68)^ 2020	Japan	Retrospective cohort study	Patients undergoing elective endovascular thoracic aneurysm repair	Surgical	Cardiovascular disease	CONUT	N/A	Single-center	Not specified	60	Overall survival; non-aneurysm-related mortality; prevalence of reinterventions	Malnutrition identified by CONUT was a negative predictor of survival prognosis
Inoue et al.^(69)^ 2019	Japan	Retrospective observational study	Patients with hip fracture after fall undergoing surgical treatment	Surgical	Hip fracture	MNA-SF, MUST, NRS 2002 and GNRI	N/A	Single-center	≥65 years	205	LOS; functional outcomes	MNA-SF showed a stronger association with functional outcomes. GNRI was significantly associated with walking speed (10 m). MUST and NRS 2002 were not associated with functional outcomes
Jayant et al.^(70)^ 2020	India	Prospective observational study	Patients with an oncological diagnosis undergoing elective surgery	Surgical	Oncologic	MUST, SGA and NRI	N/A	Single-center	≥18 years	342	LOS; postoperative complications within 30 days	SGA and MUST demonstrated good reliability for the evaluated outcomes
Jeejeebhoy et al.^(71)^ 2015	Canada	Cohort study prospective	Inpatients from medical and surgical clinics	Clinical/Surgical	Cardiovascular, neurological, gastrointestinal, respiratory diseases	NRS 2002	SGA	Multicenter	≥18 years	733	LOS; hospital readmission	SGA classification, especially category C, predicted length of hospital stay
Kalaiselvan et al.^(72)^ 2017	India	Prospective observational study	ICU patients receiving mechanical ventilation for >48 h	ICU	Respiratory failure, septic shock, neurological disorders,	m-NUTRIC	N/A	Single-center	Adults	678	ICU LOS; time off mechanical ventilation; mortality	Higher m-NUTRIC scores were associated with longer ICU stay and predicted mortality
Kang et al.^(73)^ 2020	South Korea	Prospective observational study	Patients with acute ischemic stroke admitted within 7 days of symptom onset	Clinical	Neurological disease	GNRI	N/A	Single-center	Not specified	1,906	3-month prognosis after hospitalization (mRS)	Moderate and severe GNRI risk categories were associated with unfavorable outcomes
Katayama et al.^(74)^ 2020	Japan	Retrospective observational study	Patients undergoing percutaneous coronary intervention with rotational atherectomy	Surgical	Cardiovascular disease	GNRI	N/A	Single-center	Not specified	206	Major adverse cardiovascular events, including mortality	GNRI was an independent predictor of major adverse cardiovascular events
Komici et al.^(75)^ 2019	Italy	Prospective observational study	Patients with acute myocardial infarction	ICU	Cardiovascular disease	MNA	N/A	Single-center	≥65 years	174	Mortality	MNA results were an independent predictor of long-term mortality
Kootaka et al.^(76)^ 2021	Japan	Retrospective cohort study	Patients with cardiovascular disease	Clinical	Cardiovascular disease	GLIM	ESPEN-DCM	Single-center	≥20 years	921	Mortality; physical performance	GLIM and ESPEN-DCM were significantly associated with mortality. However, only GLIM predicted low functionality
Koren-Hakim et al.^(77)^ 2016	Israel	Prospective cohort study	Elderly patients with hip fracture undergoing surgery	Surgical	Hip fracture	MNA-SF; NRS 2002; MUST	N/A	Single-center	≥65 years	215	LOS; postoperative complications; readmission within 6 months; mortality at 36 months	Only MNA-SF predicted readmissions and mortality
Lee et al.^(78)^ 2021	Taiwan	Retrospective observational study	Patients with blood cultures positive for non-albicans candida species	Clinical	Candidiasis	MUST	N/A	Multicenter	Adults	378	28-day mortality	MUST ≥2 was independently associated with a higher risk of 28-day all-cause mortality
Lim et al ^(79)^ 2014	Singapore	Prospective cohort study	Hospitalized patients (excluding psychiatric, ICU, and maternity)	Clinical	Not specified	3-MinNS	N/A	Single-center	Adults	818	LOS; hospital readmission; mortality	Higher 3-MinNS scores were associated with longer hospital stay and higher mortality at 1 and 3 years
Nascè et al.^(80)^ 2021	Switzerland	Prospective cohort study	Patients with suspected pneumonia	Clinical	Suspected pneumonia	MNA	Geriatric and functionality assessment	Single-center	≥65 years	200	Mortality	MNA significantly predicted long-term mortality (1 year)
Nishioka et al.^(81)^ 2020	Japan	Cohort study prospective	Elderly people with stroke transferred from intensive care hospitals	Clinical	Neurological disease	ESPEN-DCM	MNA-SF, MUST and GNRI	Single-center	≥65 years	420	Functionality; Discharge destination	GNRI showed predictive validity for discharge destination. MNA-SF showed fair concurrent validity but required new cutoff points
Nuotio et al.^(82)^ 2016	Finland	Prospective observational study	Patients in the perioperative period for hip fracture	Surgical	Hip fracture	MNA-SF	N/A	Single-center	≥65 years	472	Institutionalization; mortality and morbidity	MNA-SF was an independent predictor of the main outcomes studied
Oliveira et al.^(83)^ 2019	Brazil	Longitudinal study	Critical illy ill patients hospitalized fo at least 48 hours	ICU	Postoperative complications, septic shock, infection, heart problems, and gastrointestinal complications	m-NUTRIC	NUTRIC-PCR and SGA	Single-center	≥18 years	130	ICU LOS; 28-day mortality	Higher m-NUTRIC scores were associated with mortality. NUTRIC showed a positive association with SGA, With good agreement between m-NUTRIC and NUTRI-PCR
Ozkalkanli et al.^(84)^ 2009	Turkey	Prospective study	Patients undergoing elective orthopedic surgeries hospitalized >2 days	Surgical	Orthopedic	SGA	NRS 2002	Single-center	≥18 years	256	Postoperative complications; mortality; LOS	NRS 2002 better predicted postoperative complications than SGA. No significant differences were observed between tools for the other outcomes
Barbosa et al.^(85)^ 2010	Brazil	Prospective cohort study	adults hospitalized in the wards	Clinical	Not specified	NRS 2002, MNA-SF, MUST and SGA	N/A	Single-center	≥18 years	705	Complications; LOS; mortality	NRS and SGA better predict outcomes. SGA was more strongly associated with hospital LOS
Rabito et al.^(86)^ 2017	Brazil	Prospective cohort study	hospitalized	Clinical	Gastrointestinal, cardiovascular, and oncological diseases	NRS 2002	MUST, MST and SNAQ	Single-center	≥18 years	752	Morbidity Mortality	MUST showed greater identification of mortality risk (2.34-fold). All tools were sensitive for identifying prolonged hospitalization
Rasheedy et al.^(87)^ 2020	Egypt	Cross-sectional study	hospitalized	Clinical	Not specified	MNA	GNRI	Single-center	≥60 years	150	Fragility; sarcopenia	GNRI showed greater accuracy for frailty than MNA. GNRI risk categories were significantly associated with lower muscle strength
Raslan et al.^(88)^ 2010	Brazil	Prospective observational study	hospitalized	Clinical/Surgical	Metabolic, infectious, inflammatory, immunological diseases, and oncological diseases	NRS-2002, MNA-SF and MUST	N/A	Single-center	≥18 years	706	Complications; LOS; mortality	NRS 2002 and MNA-SF predicted outcomes better overall; however, NRS 2002 showed the strongest performance
Raslan et al.^(89)^ 2011	Brazil	Prospective observational study	hospitalized	Clinical/Surgical	Not specified	SGA e NRS 2002	N/A	Single-center	≥18 years	705	Complications; LOS; mortality	Combining SGA and NRS 2002 improved reliability, and both were positively associated with the evaluated outcomes
Rodrigues et al.^(90)^ 2015	Brazil	Retrospective cohort study	Patients with gynecological tumors	Clinical	Oncologic	PG-SGA	N/A	Single-center	≥18 years	146	Readmission; mortality	PG-SGA classification was consistent with the evaluated outcomes
Ruiz et al.^(91)^ 2018	Colombia	Prospective cohort study	Patients with heart and lung diseases	Clinical	Heart and lung diseases	MST	N/A	Multicenter	≥18 years	800	LOS Mortality Readmission Hospital costs	A positive MST (≥2 points) was associated with the evaluated adverse outcomes
Santos et al.^(92)^ 2017	Brazil	Cross-sectional study	Oncology patients hospitalized for at least 3 days	Clinical	Oncologic	PG-SGA	N/A	Single-center	≥20 years	333	LOS Mortality	PG-SGA was an important marker of prolonged hospitalization and higher mortality
Sanz-París et al.^(93)^ 2016	Spain	Prospective observational study	Patients with diabetes	Clinical	Diabetes	ESPEN-DCM	MNA-SF	Multicenter	≥65 years	1,014	LOS Mortality	An MNA score of 7 was associated with a 2.7-fold increase in in-hospital deaths
Söderström et al.^(94)^ 2014	Sweden	Prospective cohort study	Older adults	Clinical/Surgical	Diabetes; neurological disorders; lung diseases; rheumatoid arthritis; kidney failure	MNA	N/A	Single-center	≥65 years	1,767	Mortality	MNA predicted premature mortality
Saseedharan^(95)^ 2019	India	Prospective cohort study	Critically ill patients	ICU	Clinical and surgical	NUTRIC	SGA e NRS 2002	Single-center	N/A	348	Mortality LOS in ICU LOS	NRS 2002 and SGA correlated significantly with ICU LOS; NRS 2002 also correlated with overall hospital LOS. NUTRIC score was the best predictor of mortality
Zhao et al.^(96)^ 2020	China	Observational retrospective cohort	Patients with cardiovascular disease undergoing percutaneous coronary intervention	Surgical	Non-ST-segment elevation acute coronary syndrome	GNRI	N/A	Single-center	N/A	2,299	Mortality; Non-fatal myocardial infarction; revascularization	Lower GNRI was a significant predictor of adverse prognosis

**Table 2:** Tools evaluated in the included studies and the evidence of positive outcomes associated with their use

Tools	Mortality	ICU mortality	Overall survival	LOS	ICU LOS	Hospital readmission	Bad prognosis	Bad outcomes	Postoperative complication	Mobility	Disability	Functional outcome	Treatment toxicity
MNA-SF	x			x		x		x	x	x		x	x
MNA full	x									x			
NRS 2002	x			x			x		x				
MUST	x		x	x					x				
NUTRIC	x												
m-NUTRIC	x				x								
GNRI	x						x	x	x				
SGA	x	x		x	x				x				
PG-SGA	x			x		x							
NUTRIC	x												
MST								x					
MCRS									x				
CONUT			x				x				x		
SNAQ				x									
HFR-TNP	x												
3-MinNS	x				x								
ESPEN-DCM	x												
ANS													
PNI			x						x				
O-PNI				x									
AND-ESPEN	x					x							

ICU: Intensive Care Unit; LOS: Length of Stay; MNA-SF: Mini Nutritional Assessment Short Form; MNA full: Mini Nutritional Assessment; NRS 2002: Nutritional Risk Screening 2002; MUST: Malnutrition Universal Screening; m-NUTRIC: Modified Nutrition Risk in Critically Ill; GNRI: Geriatric Nutritional Risk Index; SGA: Subjective Global Assessment; PG-SGA: Patient-Generated Subjective Global Assessment; NUTRIC: Nutrition Risk in Critically Ill; m-NUTRIC: Modified Nutrition Risk in Critically Ill MST- Malnutrition Screening Tool; MCRS: Malnutrition-related Complications; CONUT: Controlling Nutritional Status; SNAQ: Short Nutritional Assessment Questionnaire; HRF-TNP: Royal Free Hospital-Nutritional Prioritizing Tool; 3-MinNS: 3-Minute Nutrition Screening; ESPEN-DCM: European Society for Clinical Nutrition and Metabolism Diagnostic Criteria for Malnutrition; ANS: Automated Nutrition Score; PNI: Prognostic Nutritional Index; O-PNI: Onodera's Prognostic Nutritional Index; AND-ESPEN: Academy of Nutrition and Dietetics- European Society for Clinical Nutrition and Metabolism.

### Results of syntheses

Results are presented below, highlighting clinical outcomes and the tested instruments.

The MNA and MNA-SF nutritional tools were studied in 21% of trials related to clinical outcomes,^(20,24,27,53,67,75,80,82,94)^ length of hospital stays,^(51,55)^ readmission,^(77)^ postoperative complications,^(64)^ functional outcomes,^(69)^ and chemotherapy toxicity.^(63)^ Notably, the MNA-SF screening tool demonstrated strong predictive value for mortality.^(22-24,34,53,75,77,93)^

Other nutritional screening tools also demonstrated an association with mortality in the studies included: 8% NRS 2002,^(25,30,36,48,49,88)^ 5% MUST,^(41,56,78,86)^ 5% NUTRIC,^(32,72,95)^ 5% GNRI,^(33,61,68)^ 3% m-NUTRIC,^(72,83)^ HFR-TNP,^(44)^ 1% AND-ESPEN,^(52)^ 1%MST,^(91)^ 1% NRS modified,^(40)^ and 1% 3-MinNS.^(79)^

Regarding nutritional assessment tools, evidence of mortality prediction was found for 5% PG-SGA,^(65,90,92)^ 5% SGA,^(37,65,89)^ and 1%ESPEN-DCM.^(76)^

In terms of hospital length of stay, the tools that demonstrated an association were 9% SGA,^(28,37,39,43,71,85,95)^ 3% MNA,^(51,64)^ 1% AND-ESPEN,^(52)^ and 1% O-PNI^(39)^ among the assessment tools; and 5% NRS 2002,^(39,88,95)^ 5% MUST,^(39,56,64)^ 3% MST,^(86,91)^ 1% MUST modified,^(41)^ 1%CONUT,^(39)^ 1% 3-MinNS,^(79)^ 1% SNAQ,^(86)^ and 1% m-NUTRIC^(72)^ among the screening tools.

CONUT demonstrated predictive value for postoperative complications, survival, performance, and quality of life across six studies.^(38,45,50,59,60,68)^ ANS was the only screening tool that did not show a positive relationship with clinical outcomes in the included studies.

Functional capacity was evaluated in four studies, with reported associations for MNA-SF,^(69)^ SGA,^(29)^ MUST,^(49)^ and GNRI tools.^(87)^

When stratifying the analysis for Latin populations, only 13 of the 77 studies (17%) were conducted in this subgroup, with 11 (14%) conducted in Brazil.

Of the Brazilian studies, only one was multicenter; the median sample size was 470 patients, and 7 studies were prospective. Mortality and hospital length of stay were the most frequently assessed outcomes. Among tools analyzed in Brazilian populations, SGA (assessment) and NRS 2002 (screening) were the most studied. In two studies,^(85,89)^ the combination of SGA and NRS 2002 provided more reliable results for length of hospital stay, complications, and mortality among hospitalized individuals.

Regarding tools commonly used in Brazilian hospitals, such as NRS 2002,^(10)^ MNA,^(97)^ MNA-SF,^(98)^ and SGA,^(99)^ the original validation articles did not validate these tools based on clinical outcomes. Instead, they were validated using clinical parameters such as biochemical and clinical assessments, including both positive and negative studies on the impact of nutritional therapy. These articles were considered in the PRISMA flowchart under "Identification of studies via other methods."

Concerning GLIM, it is not a screening or nutritional assessment tool but rather a set of diagnostic criteria. Six articles evaluated different criteria,^(31,46,47,57,66,76)^ all within surgical or clinical populations. Of these, 83% were conducted in Asian populations and 83% in oncological patients. The median sample size was 711 patients, and all studies were single-center. Across studies, the initial screening tool varied (NRS 2002, MUST, MNA-SF, or ESPEN-DCM). The studies also differed in the number and combinations of GLIM variables tested and added to the initial parameters. In two studies, functional measures such as walking speed and handgrip strength were included.^(66,76)^ In one study that analyzed variables independently, weight loss showed the highest correlation with survival, and low muscle mass identified by tomography was associated with worse outcomes.^(46)^

## DISCUSSION

The included studies covered populations from 31 countries and were predominantly conducted in single-center settings. Across the included evidence, 20 nutritional screening and assessment tools were evaluated. Mortality was the most frequently assessed clinical outcome, whereas hospital readmission was the least frequently reported. Overall, MNA/MNA-SF appeared to be associated with the broadest range of favorable clinical endpoints. When considering mortality specifically, MNA and NRS 2002 were most consistently associated with mortality risk. Among nutritional assessment tools, the subjective instruments PG-SGA and SGA were among the most extensively studied and showed associations with clinical outcomes.

Regarding specific populations, relatively few studies evaluated critically ill patients. This gap may reflect an important limitation of applying existing screening and assessment tools in the ICU population. The European Society for Clinical Nutrition and Metabolism (ESPEN) guideline for critically ill patients also highlights limitations of conventional screening and assessment tools in this setting, particularly because most tools were not developed using variables that are sensitive to critical illness.^(100)^ As a consensus recommendation (low level of evidence; expert opinion), ESPEN suggests considering any critically ill patient who remains in the ICU for >48 hours to be at nutritional risk (strong consensus, 96%). For nutritional assessment, ESPEN recommends detailed clinical assessment given the lack of a specific screening tool for this population (strong consensus, 100%).^(100)^ Together, these points support the need for additional prospective, multicenter studies to validate tools for critically ill patients or to develop instruments that incorporate ICU-relevant variables, given the high risk of pre-existing malnutrition and the catabolic response associated with critical illness.

In the context of GLIM, the heterogeneity of populations, variables, and tested combinations identified in this review limits direct comparisons across studies and reduces the ability to draw definitive conclusions. Similar concerns have been reported in prior literature, including a review by Fonseca et al.^(101)^ and another review by Correia et al.^(102)^ Establishing standardized research protocols will be important to improve comparability across populations and to support more consistent implementation of GLIM criteria alongside screening tools.

An additional limitation is the scarcity of studies involving Latin American populations (17%), particularly from Brazil (14%). This gap raises concerns regarding the reliability and applicability of commonly used tools in these settings, as population-specific socioeconomic and clinical characteristics may not be adequately represented in the broader evidence base.^(103)^ Future studies should prioritize Latin American populations and explicitly consider socioeconomic disparities and context-specific determinants that may influence nutritional risk and clinical outcomes.

Finally, further research is needed that prioritizes clinically relevant outcomes, evaluates distinct subpopulations, and assesses both established tools and the development of new instruments. Machine-learning approaches, supported by large clinical databases, may enable the development of simpler and more accurate tools that integrate screening and assessment functions to better guide nutrition interventions. However, clinical studies combining nutritional screening or assessment with machine learning remain limited. Notably, Muñoz Díaz et al.^(104)^ combined MNA-SF with additional features in a logistic regression model and reported improved performance using a novel variable set. Duan et al.^(105)^ used PG-SGA-based diagnoses with selected predictors in an XGBoost model to identify key predictive variables. These initiatives illustrate the potential for more efficient, concise, and accurate nutritional evaluation methods.

This study has limitations. First, the exclusive focus on hospitalized populations may limit generalizability. Second, validation studies that did not evaluate clinical outcomes were not described in detail. Third, the inclusion of "clinical outcomes" in the search strategy may have underestimated the total number of validation studies that focused primarily on diagnostic or screening performance rather than clinical endpoints.

## CONCLUSION

Overall, nutritional screening and assessment tools used in routine practice were validated many years ago, often relying primarily on subjective clinical assessments and with relatively limited reporting of clinical outcomes. In addition, most studies were conducted in single-center settings and predominantly in non–Latin American populations. Future research should prioritize multicenter designs, improve population representativeness, and incorporate clinically relevant outcomes when evaluating both established and newly developed tools.

## Data Availability

The underlying content is contained within the manuscript.
